# Diffractive Sensor Elements for Registration of Long-Term Instability at Writing of Computer-Generated Holograms

**DOI:** 10.3390/s21196635

**Published:** 2021-10-06

**Authors:** Ruslan V. Shimansky, Dmitrij A. Belousov, Victor P. Korolkov, Roman I. Kuts

**Affiliations:** Institute of Automation and Electrometry SB RAS, 630090 Novosibirsk, Russia; victork@iae.nsk.su (V.P.K.); r.i.kuts@mail.ru (R.I.K.)

**Keywords:** diffractive microstructure sensors, diffraction-based overlay, computer-generated holograms, laser writing systems, asphere testing, computer-generated holograms certification

## Abstract

The research and development of methods using of the specialized diffractive microstructure sensors embedded in the pattern of computer-generated holograms (CGH) manufactured on circular and X-Y laser writing systems is discussed. These microstructures consist of two parts: one of which is written before the CGH in the field of future hologram and the second one is written during the long-term writing of the CGH. The shift between the first and second part of the microstructure is the trace of the writing errors and allows one to determine and calculate the error of CGH fabrication along both orthogonal coordinates. The developed method is based on the principle of diffraction-based overlay with 1D and 2D built-in diffractive microstructure-sensors. Mathematical modeling and results of experimental test writings of such diffractive microstructure sensors are described. The efficiency of using these types of build-in sensors for the writing errors estimation for CGHs is demonstrated.

## 1. Introduction

The choice of exposure algorithms, technologies and materials for laser writing of precision computer-generated holograms (CGH) is largely determined by the design of the writing system used. Scanning laser lithographic systems (SLLS) described in the literature and used for the manufacturing the CGHs and micro-optical elements can be divided into two main groups: X-Y systems with a basic linear trajectory of the beam motion [1,2,3] and circular laser writing systems (CLWS) [4,5,6,7,8,9,10].

When analyzing and developing methods for writing diffractive structures, it is necessary to take into account the peculiarities of application to both groups of devices. CLWSs are most effectively used for axisymmetric diffractive optical elements, starting with relatively small-sized zone plates and circular diffraction gratings [11,12] and ending with large computer-synthesized holograms for generating an aspherical wavefront [13,14].

Long duration of large CGH writing is inherent to all the mentioned types of SLLSs. Following sources of instability can have a significant impact on the long writing process:

- Drift of various mechanical units of the system (change of air bearings gaps, thermal expansion) due to changes in their temperatures [15];
- Focused laser beam drift (due to instability of beam pointing diagram and optical axis of focusing lens moved by the electrodynamic or piezoelectric actuator of the autofocus system);
- A change in the wavelength of a laser interferometer that controls linear displacements, which can be not fully compensated when using the formula for dependence of refractive index on the temperature, pressure and humidity of the ambient air [16];
- Displacement of the hologram substrate on the spindle faceplate or linear displacement table due to sharp acceleration or vibration at insufficient mechanical fixation.

Therefore, the problem of recording the long-term instability of the writing beam coordinates for each manufactured CGH is critical. Positioning errors occurring during writing can be used as evidence of the quality (certification) of a manufactured CGH or to account for errors in processing interferograms in optical inspection systems for aspherical surfaces using a reference wavefront generated by the CGH.

Devices built in the SLLS for measuring the coordinates of the writing beam relative to the substrate with the light-sensitive material cannot, as a rule, be used to fix current manufacturing errors, since their own drifts cannot be detected in this case. An example is the change in the wavelength of a laser interferometer measuring linear shift due to a change in air pressure or temperature. Nevertheless, local holding errors of the positioning system, which are expressed in the deviation of the measured position from the preset one, can certainly be recorded using a standard coordinate measuring device. This is especially the case when the positioning error due to vibrations is outside the specified acceptable limits.

A typical solution for controlling the errors during or after the end of the manufacturing process is to build various marks in the structure to be manufactured, which act as sensors for coordinate errors that occurred at some moment in the writing process. Over the decades of the development, manufacture and operation of the circular laser writing systems, the staff of the IA&E SB RAS has proposed and implemented various types of fiducial marks reflecting both the specifics of laser writing in a polar coordinate system and the specifics of typical manufactured diffractive elements. [15,17,18,19,20,21,22,23].

Studies of the errors for SLLS operating in Cartesian coordinates were also carried out [2,3,24]. Unfortunately, we didn’t find out published results for commercially available SLLSs at present. 

We propose in this article the approaches based on diffractive sensor elements (DSE) which can be used by developers and users to test the stability in time of both experimental and commercially available precision SLLS. This article discusses various configurations of the DSE, the results of modeling their diffraction efficiency, as well as the experimental results on writing and characterizing the DSE.

## 2. Materials and Methods

### 2.1. Laser Writing Systems: Specificity and Errors at Writing of CGHs

SLLSs operating in Cartesian or polar coordinates form an arbitrary (having non-circular and nonlinear elements) pattern with approximately the same errors at the same level of systematic errors. The process of CGH writing by means of such a system introduces errors into the formed structure. These errors have two generally independent components:

- The difference between the assumed and real coordinates of the center of the elementary cell of the CGH pattern;
- Differences in the shape and size of the calculated and actually written elementary cell (size error).

The first component depends on the positioning error of the laser beam with respect to the origin of coordinates and, in turn, the origin of coordinates with respect to the substrate, which can be displaced on the linear stage or rotated spindle faceplate during long-term writing. The second error is caused by changes in the size of the trace formed by the laser beam. Both of these components determine the position of the zone boundaries when writing binary CGHs. The local distortion error ζ (in the direction perpendicular to the boundaries of the diffractive zones) in the microstructure of binary CGH is determined by averaging the positions of the inner and outer boundaries of the zone (ζ_in_ and ζ_out_):

ζ = (ζ_in_ + ζ_out_)/2. (1)

The local error in the distortion of the diffractive structure leads to an additional phase shift of the wavefront of the light flux passing through the fabricated CGH. This local wavefront aberration A can be defined as [23,25]:

A = −m_D_·λ·ζ/(T·m_R_), (2)

where m_D_—designed diffraction order, m_R_—diffraction order at reconstruction, λ—wavelength and T—local period of diffractive zones of the CGH. Below we will consider some specific sources of local CGH errors when writing on various types of SLLS.

**Figure 1:**
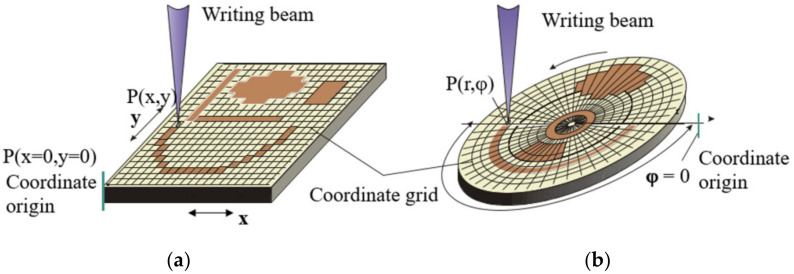
Scanning of writing beam and writing of arbitrary pattern by X-Y SLLS (**a**) and CLWS (**b**).

Figure 1 shows examples of the formation of the arbitrary patterns by means of both SLLS types. Figure 1a shows that the origin of coordinates for of the X-Y SLLS can be set arbitrarily. However, after the origin setting is made, it must also be stationary relative to the CGH blank. As it is evidently from Figure 1b the origin of coordinates for the CLWS should be aligned with the spindle rotation axis in order to avoid distortions of the formed pattern. The problem is how to check that the origin of coordinates during writing process is still in the same place as at the beginning. When several small patterns are formed on one blank, this is not so important if their relative position is not critical for task specification, and the drift of the origin of coordinates during the writing of one pattern is negligible. When writing large CGHs, where the mutual positions of all diffactive zones are set and very important, it is necessary to track that the origin of each axis of writing beam motion is associated with a specific place. The task can be solved by preliminary formation of sensor elements or alignment marks on the blank of the CGH. When using thermochemical [26,27] or thermo-structural [28] writing, the sensor element can be written right before fabrication of main hologram by burning, for example, a ring in the center of the CGH blank when using CLWS [17]. At usage of photoresist as recording material, the formation of the sensor element requires a separate complete technological cycle from applying the photoresist to etching the mask layer which is the carrier of the sensor element [21].

**Figure 2:**
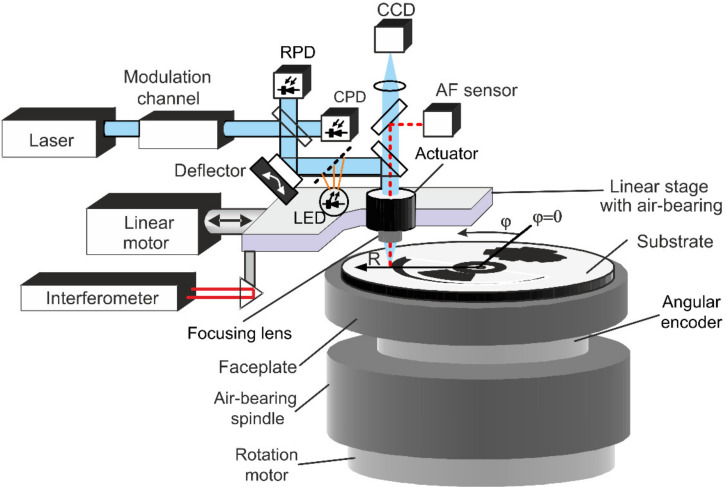
Simplified diagram of the CLWS (RPD—reflection photodetector, CPD –photodetector for laser beam power calibration, LED—LED illuminator for observing the surface with CCD-camera).

An equally important issue is the method of reading the position of the DSE. In XY SLLS, an optical microscope built into the system is usually used for measurement of the pattern made in previous technological step. For CLWS (Figure 2), this method is not suitable because it is required to stop the spindle rotation. The axis of the spindle rotor usually deviates from the vertical after it stops. Photoelectric readout of the intensity of the light beam reflected from the writing material makes it possible to read with high accuracy the marks written on the writing material. A simplified CLWS diagram on Figure 2 shows reflection photodetector RPD used for monitoring a reflection of the writing material. In the reflection measurement mode, the attenuated writing beam is used as a probe beam. Reflection measurement is performed synchronously with the spindle rotation and the movement of the linear table with the focusing lens. This makes it possible to map the local changes in the reflection of the writing material applied to the substrate. In this case, the position of the marks by the Foucault knife method along the radial coordinate is measured with a spatial resolution at the nanometer level, despite the fact that the diameter of the focused probe beam does not exceed 700 nm. Such spatial resolution in measurements is significantly better than that observed with an optical microscope in the visible range, where the resolution is at the level of the light wavelength and is not increased by increasing the number of camera pixels.

For the problem of monitoring surface structures, an important role is played by the choice of recording materials, which can be divided into photo- and thermosensitive. Thermosensitive materials have several advantages over photosensitive media:

- Low requirements for the quality of the focusing optics and the presence of scattered light in the optical channel;
- A slight increase in the power required for writing, with an increase in scanning speed;
- The nature of the action of the laser beam on the recording material is close to the threshold one and makes it possible to obtain the width of the written track much less than the diameter of the writing spot.

Its most important term for thermo-sensitive writing material is usually a noticeable change in light reflectance. A change in reflection from a photoresist after exposure can only be reliably detected if it melts or evaporates, resulting in it being overexposed in a wide area around the altered region.

This change in light reflectance is convenient to use for forming a circular mark intended to measure the position of the spindle rotation axis relative to the origin of the radial coordinate in the CLWS. A small ring (with a radius of 10–100 microns) is written on a thermo-sensitive film sputtered on the CGH substrate around the rotation center and its radii are measured to the left and right of the rotation center using a photoelectric scanning [17,23]. The change in reflection occurs due to the melting or evaporation of the film. Scanning photoelectric readout is carried out using the RPD photodetector (Figure 2).

The probe spot is shifted to the first outer radius *r*_1_ = *r*_0_ + *S/2*, where *S* is the scanning range, and scanning is performed with a step of radial movement of 30–50 nm towards the center. The scanning range is usually 3–6 µm. Then scanning is performed in the range *±S/2* relative to the opposite radius −*r*_0_. After searching for the coordinates of points with minimal reflection (*r*_1_ and *r*_2_), the error of the coordinate of the center of rotation is determined through the expression *Δr* = (*r*_1_ − *r*_2_)/*2*.

The simplest type of marks allows solving a number of tasks on a CLWS connected with the current determination of coordinate errors and the forecast of the direction and magnitude of the system drift. However, such a mark cannot be analyzed after finishing writing and removing the CGH substrate from the spindle faceplate. From the point of view of certification of the fabricated CGH, it is desirable to have some built-in sensors, which can be checked by the customer of the CGH, if necessary.

### 2.2. Method of Built-In Marks (Sensor Elements) for Manufactured CGH Certification

From the above, it follows that minimization, registration and inspection of errors at CGH writing are key tasks in its manufacturing. In particular, the solution of the tasks is critical at manufacturing CGHs used for generating reference wavefront at optical testing of aspherical telescope mirrors when wavefront error of not more than λ/20 is required for the holograms which are a few hundred millimeters in diameter [29]. 

Since the creation of the first version of CLWS, the staff of the IA&E SB RAS have proposed, developed and implemented a series of techniques to ensure and confirm the required accuracy of the CGH writing. These techniques can be divided into three main categories:

- Techniques used before writing to minimize errors due to design features of positioning subsystems, beam power control, fixing an optical blank on a spindle, etc.;
- Techniques used during CGH writing for dynamic control and correction of the errors arising, for example, with a change in the laser wavelength of the interferometer controlling the radial coordinate;
- Final error inspection after finishing the CGH writing.

One of these techniques is based on usage of built-in marks to control manufacturing errors after the finishing the manufacturing process. They act as sensors for the errors in the CGH microstructure that occurred at some point in the writing process. 

The advantages of this technique are its ease of implementation, versatility for both circular and X-Y SLLS. However, the most important thing is the ability for the customer of a particular CGH to re-measure the built-in marks to confirm the quality of the product using the methods described below. Built-in marks, which essentially act as sensors for writing errors, are written in the CGH pattern and are an integral part of the CGH. The weak side of this technique is that it allows one to register only a part of the errors that are inherent to the writing systems. The first chapter of this article provides the rationale for these restrictions.

Research, development and implementation of the built-in mark technique are important tasks for CGH manufacturing. Calculation of the errors (that occurred during writing on such structures) can be used as evidence of the quality (certification) of the manufactured CGH. They can be also taken into account at processing of interferograms obtained during testing an aspherical surface using the reference wavefront generated by the CGH.

#### 2.2.1. Built-In Marks (Sensors Elements) Based on Image Analysis

**Figure 3:**
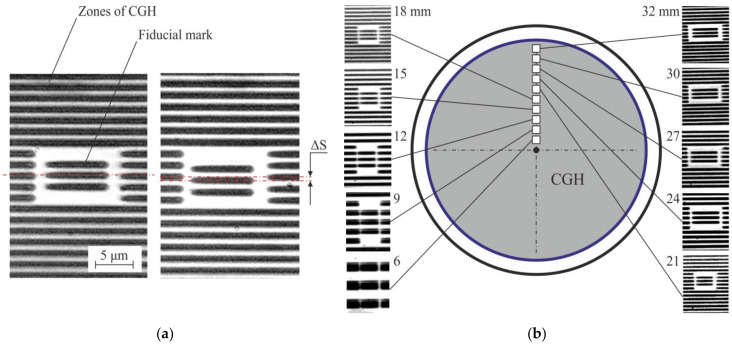
Microphotographs of fragment of amplitude CGH (chromium on glass) with written mark without error (**a left**) and with error (**a right**), ΔS = 0.4S≈0.7 μm. Layout (**b**) of marks in the pattern of rotationally symmetric CGH with 64 mm diameter.

The idea of using marks built into CGH pattern was first realized for CLWS. At the beginning, the marks did not have their own constant structure, but were actually a part of the CGH. Small sections of CGH were written before writing process at certain radii [22]. The sections were placed in a special window of a slightly larger size so that the mark would not merge with it when writing CGH. This made it easy to find the marks on the CGH pattern when photographing and taking measurements. Figure 3a shows examples of photographs of such marks and their schematic arrangement on a CGH substrate (Figure 3b). This type of marks has proven itself well at the beginning of use, but there are several significant disadvantages at once:

- They can only be effectively used for axisymmetric CGHs.
- Quite large size of the window of a built-in mark depends on its position on the CGH pattern;
- With an increase in the size of the zones, the accuracy of estimating the shift of the mark relative to the main structure of the CGH decreases significantly;
- Based on these built-in marks, one can fairly accurately determine the error on the radial coordinate. However, the estimation of the error for the Y coordinate is difficult, since this error manifests itself on them as a shift of the structure inside the mark window along the diffractive zones (for example, Figure 3a).

To universalize the processing of marks, their size was fixed at the level of about 30 × 30 μm. It made it possible, on average, to obtain 3 diffractive zones of CGH in the window of the built-in mark. However, this type of marks has become useless for wide diffractive zones.

**Figure 4:**
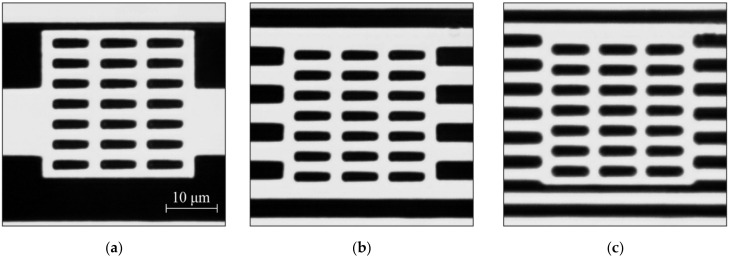
Microimages of the marks written at different hologram radii: 6 mm (**a**), 21 mm (**b**), 33 mm (**c**).

For this reason, a new type of built-in marks has been implemented. The type of marks has its own internal structure. Figure 4 shows micrographs of these types of the structures generated independently of the CGH structure. In this case, the internal grid is written before the start of CGH writing and the two framing grids during CGH writing upon reaching the writing radius on which the first part of the mark was written. This type of marks made it possible to overcome several shortcomings of the previous version at once: it can be used for any types of CGH; a fixed size of the built-in mark window (30 × 30 μm—allows writing 6–7 grating periods in the window, which increases the accuracy of the error estimation); constant measurement accuracy of the CGH writing error (~100 nm).

Consequently, for this type of built-in marks, the main significant drawbacks are only the inability to estimate accurately the CGH writing error arising in the direction orthogonal to the radial coordinate.

**Figure 5:**
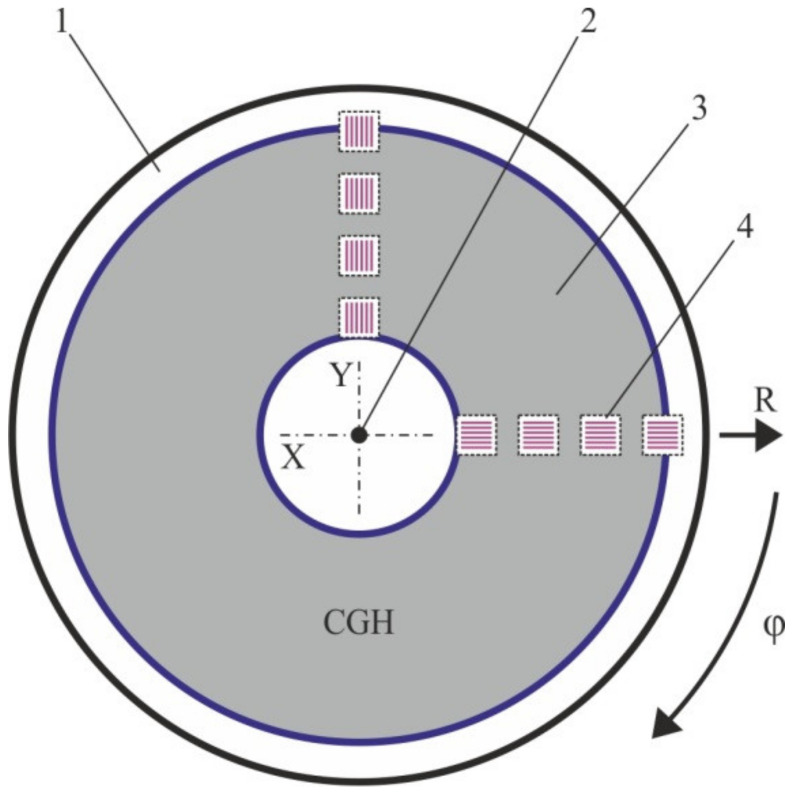
Layout of two chains of coordinate marks with 90 degrees shift for circular writing systems. 1—optical substrate, 2—axis of rotation of the optical substrate, 3—CGH, 4—small window areas with coordinate marks.

It has been also suggested to write two chains of marks in the CGH pattern rotated by 90 degrees. Figure 5 demonstrates an example of such an arrangement of the written marks. This approach made it possible to visually record not only the drift of the radial coordinate (which is recorded on both series by the same way) but also the error associated with the drift of the substrate during writing. In this case, on the first chain, the shift of the substrate along the 0-th angular position of the spindle (the beginning of the revolution) is recorded. On the second chain of the marks, respectively, a shift will be recorded in the perpendicular to the first direction.

Due to the growing requirements for the accuracy of manufacturing and certification of CGHs, as well as an increase in their number, the laboriousness of their photographing and processing has become a limiting factor.

**Figure 6:**
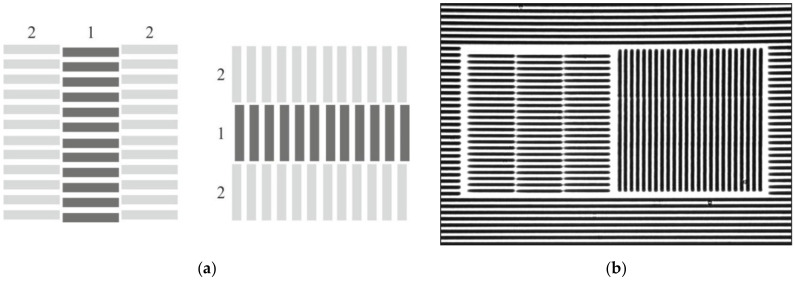
General configuration of two-coordinate built-in marks (**a**) and micrograph of a real mark (**b**).

In this regard, we have proposed a new type of two-coordinate built-in marks and a technique for their automatic measurement, based on the interferogram processing algorithm [30]. Using this new type of the built-in marks, it was planned not only to automate the measurement process, but also to significantly increase its accuracy (estimated to be up to 30–50 nm). Figure 6 shows a general diagram (a) and photomicrograph (b) of this type of built-in structures.

**Figure 7:**
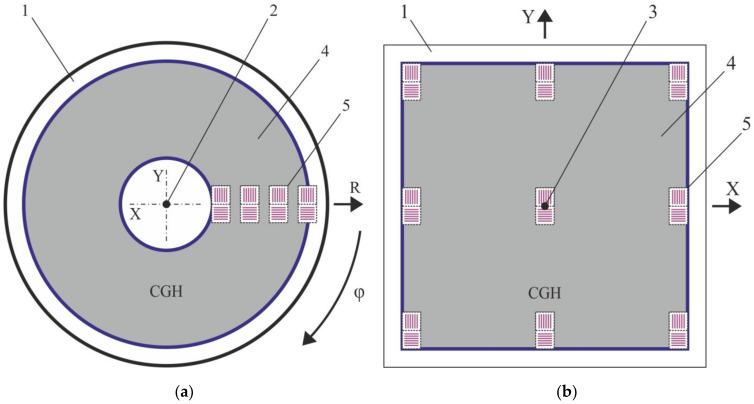
Layout of two-coordinate marks for writing systems: circular (**a**); X-Y (**b**). 1—optical substrate, 2—axis of rotation of the optical substrate, 3—origin of coordinates for X-Y laser beam positioning system, 4—CGH, 5—small window areas with coordinate marks.

In contrast to the previous type of the marks, the use of two-coordinate marks allows writing only one chain of the auxiliary structures in the CGH pattern. It allows one to determine the error along both coordinates using one double mark at once. Figure 7a shows a variant of the layout of two-coordinate marks for a circular writing system. It should be also noted that these marks can be used for the same purpose for X-Y SLLS. Figure 7b shows a variant of layout of such structures in the CGH pattern when it is written on the X-Y system.

The following conclusions were made from application of the proposed two-coordinate marks and the algorithm for their processing: the formed gratings make it possible to record clearly the CGH writing error, but it was not possible to obtain a stable measurement accuracy. The proposed method turned out to be sensitive to the choice of the initial calculation parameters and the quality of the mark microimage. Basically, this is due to the fact that the written structure is not an “ideal” interferogram, and an adapted algorithm for processing interferograms is used to calculate the error. Moreover, to obtain more or less satisfactory results for the measurement accuracy of the error (up to 30–50 nm), it is necessary to have at least 20 grating periods in the mark. In this case, it is necessary either to make the window large (at least 50 × 100 μm), or to reduce the period of the structure, that is technologically quite difficult to implement in practice.

Based on the obtained results, it was proposed to use a new type of built-in marks for evaluating CGH errors—diffractive sensor elements.

#### 2.2.2. Diffractive Sensor Elements

**Figure 8:**
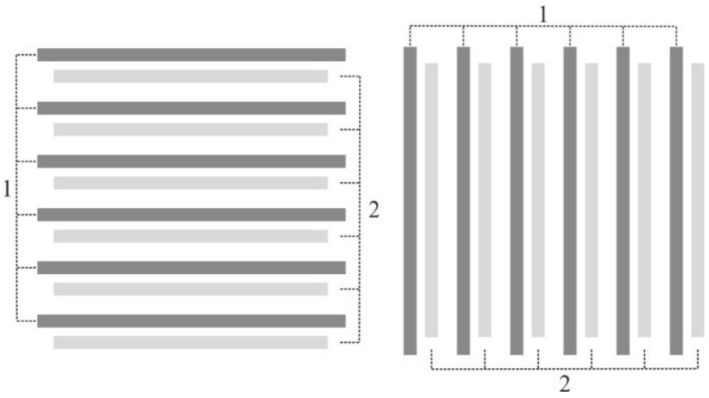
Configuration of twin 1D diffractive sensor element.

Initially, a variant of a one-dimensional diffractive sensor element (DSE) was proposed and investigated [31]. 1D DSE is two overlayed diffractive gratings with the same duty cycle and period. Figure 8 shows a configuration of a twin 1D DSE, which allows one to determine the error at once by two coordinates of the SLLS. First couple of the gratings (1 in Figure 8) is written before CGH writing at specified radii with a specified period, and the second one (2 in Figure 8) is written with a predetermined shift value relative to the first during CGH writing upon reaching the specified radii.

This type of the mark is very similar in meaning to the structures used in diffraction-based overlay metrology (DBO) developed for microelectronics manufacturing. It helps to align layers of integral circuits with nanometer precision [32,33,34]. The main difference between the use of such structures to determine the CGH errors is the need to write a sufficiently large number of DSEs in the CGH pattern and the fact that, in contrast with DBOs, DSEs are formed in one layer by using the same technology. The latter difference leads to a different simulated and experimentally investigated configurations. 

To determine the CGH writing error using microstructures of this type, it was proposed to use the method of optical diffractometry. The shift of the written gratings relative to each other will lead to a redistribution of the light intensities between the diffraction orders. Thus, by measuring the intensities of the diffraction orders, when the formed two-coordinate sensor is illuminated with a probe laser beam, it is possible to determine the real value of the shift of the gratings relative to each other which is equal the local CGH structure error along one of the coordinates. The number of periods in the grating can be reduced to 5–10. In addition, the process of measuring and calculating the CGH errors can be fully automated. The confirmed achievable sensitivity of measuring the errors in manufactured CGH using 1D DSE is approximately 5–10 nm in the range of 100–150 nm shift from the “ideal” (written without errors) local position of the CGH structure. The detailed results of the study of 1D DSE structures and the results of experimental writings of 1D DSE and their measurements are published in [35], as well as in the article accepted for publication.

For more optimal use of these types of diffractive microstructures, it was proposed to investigate the possibility of studying 2D DSE structures. When using the 2D version, the window size for the diffractive sensors in the CGH pattern can be reduced by 2 times to the size which is completely invisible at interferometric testing with the CGH. 2D DSE structures make it possible to determine the error in two coordinates at once in one measurement by a diffractometer. It was also assumed that the accuracy of determining the errors of the manufactured CGH using 2D DSE will be similar to the 1D DSE. Moreover, for such a variant of the layout of diffractive sensors, the automation of measuring the error of the manufactured CGH can be significantly optimized in time.

#### 2.2.3. Mathematical Modeling of the Diffractive Sensors

Mathematical modeling of diffraction on the proposed structures was carried out to estimate the efficiency and accuracy of the method for determining the error in CGH fabrication using 1D and 2D DSEs. Modeling was realized using the Grating Diffraction Calculator (GD-Calc) [36], which is a set of libraries for calculating the diffraction efficiency (DE) in the MATLAB software package [37]. The algorithm of the GD-Calc is based on a generalized version of the theory of rigorous coupled-wave approximation (RCWA), which allows one to calculate the diffraction efficiency within the framework of a rigorous electromagnetic theory. 

The following structure parameters were used in the modeling process: the grating period (for each of the two gratings making up the DSE structure)—5 μm, the grating structure material—chromium (n = 3.1395; k = 3.3152), the height of the Cr structure protrusions—50 nm, the line width the first and second gratings are 1 μm, the substrate material is fused silica (n = 1.4570), the probe beam wavelength is 633 nm. During the simulation, the width of the gap between the grating lines varied in the range from 0.1 μm to 1.5 μm. With a line width of 1 μm and a gap between them of 1.5 μm, the modeled structure corresponds to a grating with a period of 2.5 μm.

**Figure 9:**
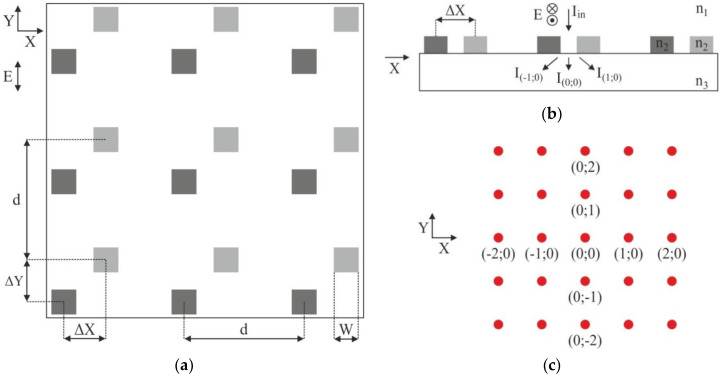
Geometry for 2D DSE modeling: layout of the gratings (**a**); sectional modeling geometry (**b**); diffraction orders notation (**c**).

Figure 9a shows the geometry of a 2D DSE simulation. The dark gray color denotes the 2D grating with period d formed before the writing of the CGH, and the light gray color denotes the shifted grating formed during the writing process. The feature size W in the calculations was 1x1 µm. Vector E indicates the direction of polarization of the probe laser beam, which was used in calculating DE. Figure 9b shows a cross-sectional geometry of the simulation (side view). For 2D DSE, the diffraction efficiency (DE) of the diffraction orders was calculated in the substrate material (i.e., without taking Fresnel losses upon reflection of radiation from the back side of the substrate into account—when modeling 1D DSE, reflection from the substrate was taken into account). Figure 9c shows the designation of the key diffraction orders in the formed diffraction pattern. Since the diffraction pattern for 2D DSE diverges in space along the X and Y axes, the diffraction orders (DO) are numbered by two values m_1_ and m_2_, each of them indicates a serial number along the X and Y axes, respectively.

Experimental writings of test DSE structures were carried out on two different writing systems: CLWS300IAE and X-Y SLLS from HIMT—DWL66+ [38]. The microstructure is made in “positive” (which corresponds to the general parameters in the description of modeling) on the first laser writing system. On the second system, the structure turns out to be “negative”. For this reason, additional modeling was carried out for “negative” structures. In this case, those parts of the structure that were protrusions on the substrate were presented as holes with the same parameters.

## 3. Results

### 3.1. Results of Numerical Simulation of 2D Diffractive Sensors

The results of the simulation showed that the DE of the 0th order is practically independent of the shift of the centers of the gratings forming the 2D DSE relative to each other. The deviation of the DE for the 0th order is less than 0.2% in the entire simulated range of shifts ΔX and ΔY. The energy redistribution upon the shift of the centers of the 2D DSE structures is pronounced in the next orders of diffraction. First and second orders are proposed to be used for monitoring using the optical diffractometry.

**Figure 10:**
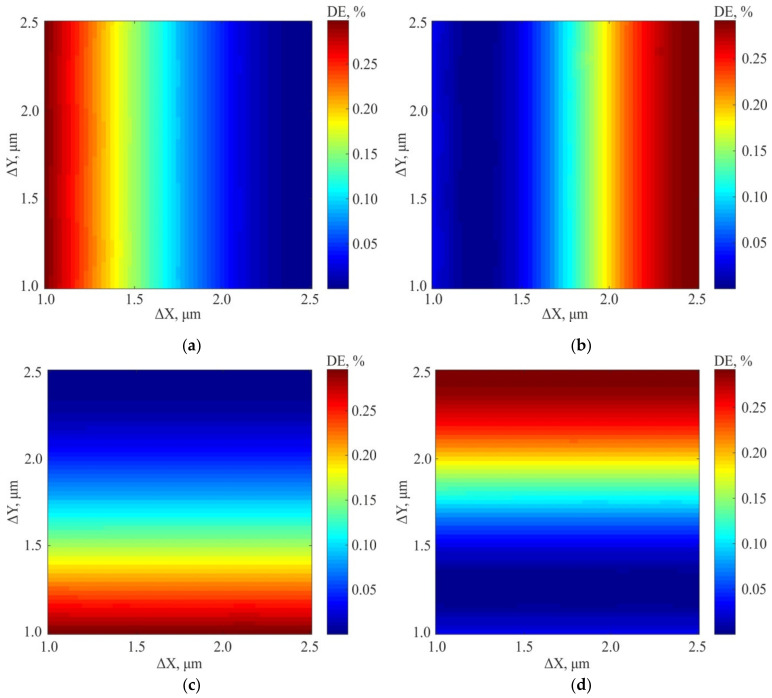
Diffraction efficiency distribution maps for positive amplitude structure of 2D DSE: Diffraction order (1;0) (**a**); Diffraction order (2;0) (**b**); Diffraction order (0;1) (**c**); Diffraction order (0;2) (**d**).

The obtained simulation results show that the diffraction efficiency of the orders (1; 0) and (2; 0) lying on the X axis is practically independent on the center shift for the second inscribed 2D grating relative to the first one along the Y axis. However, when the center is shifted along the X axis, a redistribution of energy between the first and second diffraction orders is observed (Figure 10a,b). This is also true for diffraction orders (0; 1) and (0; 2) lying on the Y axis, for which the energy redistribution is observed depending on the ΔY shift (Figure 10c,d), but their DE is practically independent on ΔX shift. Thus, the energy redistribution is observed at measuring the diffraction efficiency of four diffraction orders. It can be used to estimate the shift along both coordinates happened during writing. Qualitatively, the graphs of the redistribution of the diffraction efficiency between the DO for the ΔX and ΔY shifts coincide, and their quantitative discrepancies are insignificant. In this regard, below the calculated data for the diffraction efficiency of the test gratings will be presented for the shift of the structure along one coordinate (ΔX).

**Figure 11:**
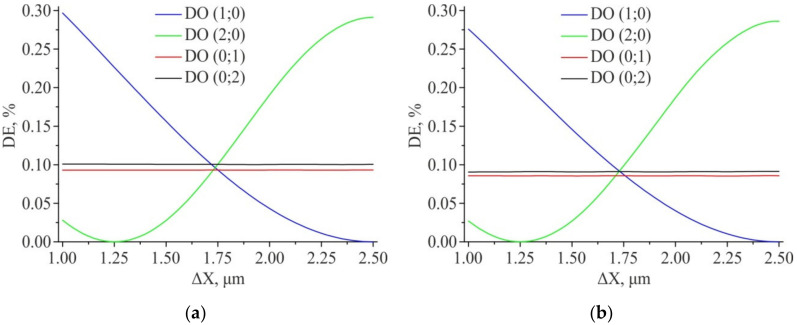
Results of modeling the diffraction efficiency for the amplitude structure 2D DSE (ΔY = const = 1.75 µm): “Positive” structure (**a**); “Negative” structure (**b**). Feature size—1 µm × 1 µm.

Figure 11 shows the graphs of the DE of the analyzed orders, depending on the value of the ΔX shift and at a fixed value of the shift of the structure ΔY = 1.75 μm (the center of the simulation range). The obtained results show that the equilibrium DE of the first and second diffraction orders for “positive” amplitude structures is achieved at ΔX ~ 1.737 μm, and for “negative” ones at ΔX ~ 1.729 μm. That is near from the center measuring range.

**Figure 12:**
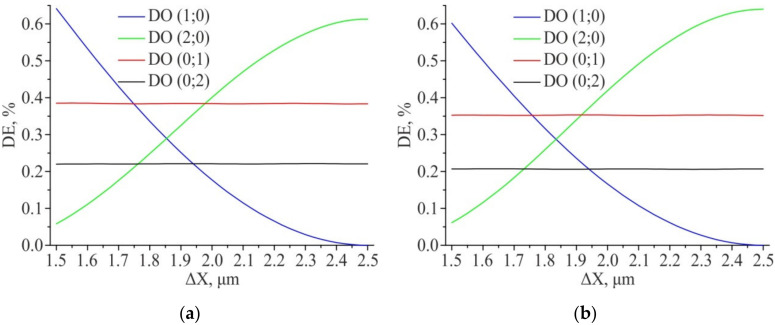
Results of modeling the diffraction efficiency for amplitude 2D DSE (ΔY = const = 1.75 μm): “Positive” structure (**a**); “Negative” structure (**b**). Feature size—1.5 µm × 1.5 µm.

Figure 12 shows the results of the diffraction efficiency modeling for amplitude 2D DSE at feature size equal to 1.5 µm. An increase in the feature size leads to a redistribution of energy from the 0th DO to the surrounding ones. It is interesting to note that the point of equality of the diffraction efficiencies for the 1st and 2nd DOs (the equilibrium point) with an increase in the feature size lies in the region of the second grating center shift relative to the first one along the X axis by ΔX ~ 1.85 μm (for the shift along the Y axis, the equilibrium point is also in the region ΔY ~ 1.85 μm). Thus, if an increase of feature size is used to increase the energy in the first and second DOs, one should remember that the equilibrium point could shift. All this suggests that the equilibrium point depends on the type of the 2D DSE structure and on its feature size.

### 3.2. Experimental Writing of DSEs

**Figure 13:**
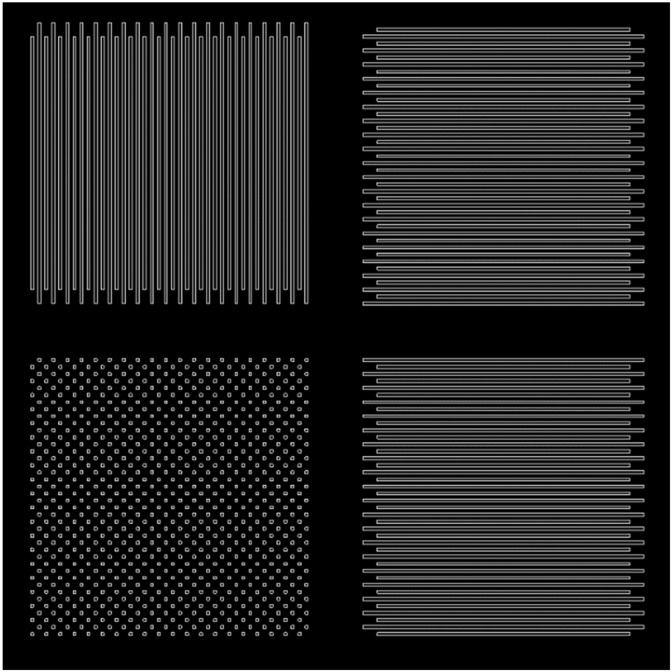
An example of a block with 1D and 2D DSE with 20 periods of diffraction gratings, a base period of 5 μm, a feature size of 1 μm and the shift of the second set of gratings ΔX and ΔY of 2.5 μm. Total block size approx. 200 × 200 μm.

A series of test DSE structures was written for comparing the simulation and experiment. The structure was based on one block of three 1D and one 2D DSEs with 40 periods of diffraction gratings with designed period of each part of the gratings of 5 μm and a designed feature size of 1 μm. The size of one 1D or 2D DSE was 200 µm. The total block size was approximately 400 × 400 μm. This size was chosen for the convenience of subsequent measurements of experimental structures on a diffractometer. Figure 13 shows a diagram of such a block with 20 periods and a total size of about 200 × 200 µm.

For each block, it was possible to set its own individual ΔX and ΔY shifts for the second part of the DSE structures. Then, DSEs of 10 blocks were formed with a given set of the shifts in X and (or) Y with the following values in μm: 2.5, 1.25, 1.5, 1.6, 1.7, 1.75, 1.8, 1.9, 2.0, 2.25. From 4 DSEs with different shifts options (either only along X or Y, or shifts along X and Y), blocks of 4 DSEs were fabricated. Each of the blocks was written several times with different parameters of the laser exposure and with the correction of the designed feature sizes after etching. The feature size near the given 1 μm value was used in the performed mathematical modeling.

The tests were written by means of two systems: CLWS300IAE (writing laser wavelength—532 nm, focusing spot diameter—700 nm, focused laser beam power—20–40 mW, spindle rotation speed—720 rpm, used radial range 9–28 mm, recording material—40–50 nm Cr film) and DWL66+ (writing laser wavelength—375 nm, laser power—45–48 mW, transmission coefficient of the filter—25%, 4mm writing head (0.6 micron resolution)).

The DSE data description was made as set of rectangles at absolute coordinates in Drawing eXchange Format (DXF). These data were used for both CLWS300IAE system and DWL66+. DXF data was translated to the internal *R-ϕ* vector format for CLWSIAE300 using special developed the rasterizer software. The rasterizer has some special input parameters (for example: radial writing step, radial spot size, angular spot size and other). These parameters were used to correct writing process to obtain necessary structure (initially specified in absolute coordinates). Standard software package of the DWL66+ system was used to write the DXF data on it.

The structures written on CLWS300IAE by the direct thermochemical writing on Cr film were “positive” according to terms above. The structure written on the SLLS DWL66+ by means of a photoresist coated on Cr film were “negative”.

The method of optical diffractometry was used for measuring the characteristics of the written experimental DSEs. The optical stand was assembled on the basis of a standard diffractometer scheme. A laser beam with a wavelength λ = 633 nm falls on the lens (F = +10 cm) which focused it on a sample with DSE. The spot diameter at the waist was ~100 μm. The sample was located on a movable X-Y table with a through window for passed diffraction orders. The laser beam power meter recorded the intensity of the diffraction orders.

**Figure 14:**
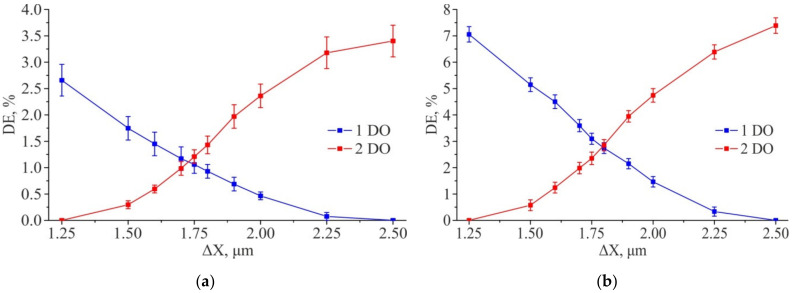
Measurement of diffraction efficiency of experimental 1D DSE structures written: on CLWS300IAE (**a**); on DWL66+ (**b**).

Figure 14 shows the results of measuring the DE of the 1st and 2nd diffraction orders for the 1D DSEs. The equilibrium DE of 1st DO and 2nd DO is in the region of shift ΔX ~ 1.75 μm, which coincides with the simulation result. The results obtained indicate the applicability of using 1D DSE structures for monitoring the coordinate errors with a sensitivity of at least 5-10 nm in the range of 100 nm shift from the “ideal” position, both for circular and X-Y SLLS.

**Figure 15:**
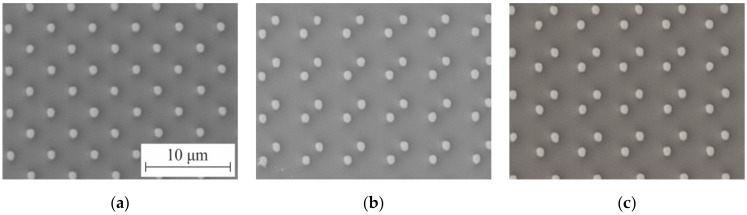
SEM microimages of 2D DSE structures written on the CLWS300IAE: test structure with shift of 2.5 μm (**a**); with shift of 1.8 μm (**b**); with shift of 1.6 μm (**c**).

**Figure 16:**
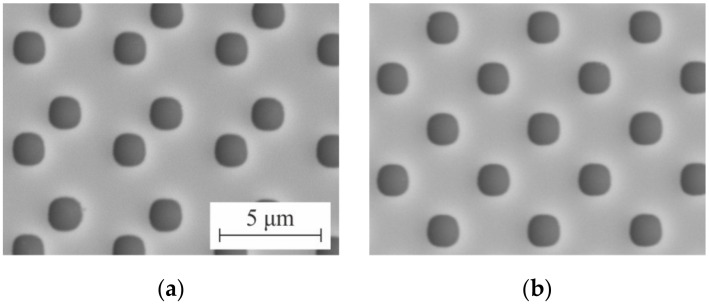
SEM microimages of 2D DSE structures written on DWL66+: general view of the test structure with shift of 1.6 μm (**a**) and with shift of 2.5 μm (**b**).

Figure 15 shows microimages of the 2D DSE structures written on the CLWS300IAE and obtained with SEM. These structures are Cr pillars on a fused silica substrate. The measured size of the written structures forming the test 2D gratings in the experiments were 1.1 × 1.1 μm. The structures written on the X-Y SLLS DWL66+ are transparent holes formed in the chromium film. The dimensions of the formed 2D gratings of the holes were ~1.3 × 1.3 μm in the experiments. SEM microimages of these structures are depicted in Figure 16.

**Figure 17:**
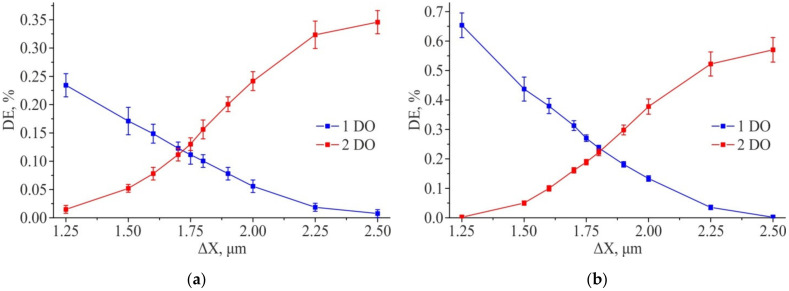
Measurement of diffraction efficiency of the experimental 2D DSE structures written: by CLWS300IAE (**a**); by DWL66+ (**b**).

Figure 17 shows the results of measuring the diffraction efficiency of the 1st and 2nd diffraction orders. The results obtained show good qualitative agreement between the experimental results and the results of the numerical simulation (Figure 11a corresponds to 17a, 12b − 17b). The shift of the equilibrium points of the diffraction efficiencies in Figure 17b to the region ΔX ~ 1.80 μm is due to the fact that the size of the DSE structures written on DWL66+ was ~1.3 μm instead of 1 μm (this also confirms the simulation result presented in Figure 12). Thus, the obtained results indicate the applicability of using 2D DSE structures for monitoring coordinate errors with sensitivity of at least 5–10 nm in the range of 100 nm shift deviation from the “ideal” position at once along two orthogonal coordinates, both for circular and XY laser writing systems.

## 4. Discussion and Conclusions

Fairly good agreement of the experimental graphs in Figure 17 with the simulation results in Figure 11 and Figure 12 does not give a direct answer to the question of how to more reliably use this data to determine the shift between overlayed gratings. From our point of view, the normalized difference in diffraction efficiencies for the 1st and 2nd orders is most convenient for using as a parameter for estimating the results of DSE measurements

ΔDE12=DE1−DE2DE1+DE2.

**Figure 18:**
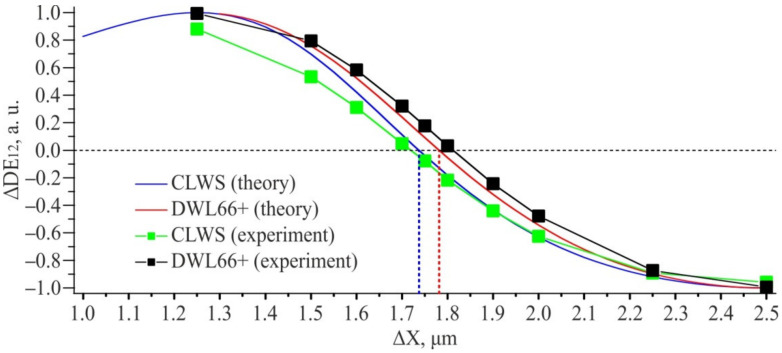
The ratios of the DE difference for diffraction orders (1; 0) and (2; 0) to their sum.

Figure 18 shows the graphs ∆*DE*_12_ for diffraction orders (1; 0) and (2; 0). The theoretical calculation was made for structures written on CLWS with W = 1 µm, and for structures written on a DWL66+ with W = 1.3 µm. The obtained experimental and calculated data are in good agreement with each other. At the same time, it is important to notice that the equilibrium point shifts with a change in the feature size. The parameter ∆*DE*_12_ changes by ±25% of the maximum possible value in the error range of ±100 nm which is considered as admissible for CGH. The error of DSE method should not exceed 20 nm even taking into account the 5% error typical for photoelectric measurements.

**Figure 19:**
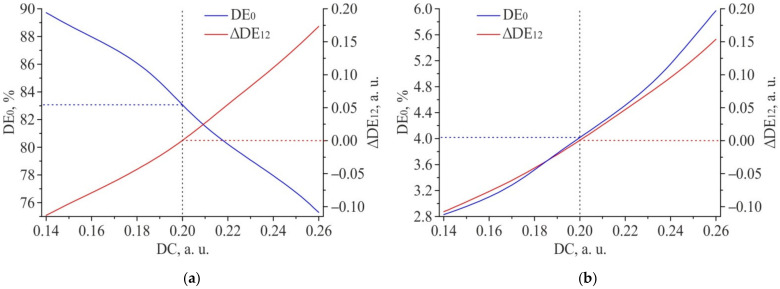
Influence of the feature size in the 2D DSE on the DE of the 0th diffraction order and parameter ∆*DE*_12_: “Positive” 2D DSE (**a**); “Negative” 2D DSE (**b**).

In addition, it can be noted that a change in the feature size of the 2D DSE structures affects the diffraction efficiency of the 0th diffraction order. Figure 19 shows the results of modeling the dependence of DE for 0th diffraction order (*DE*_0_) on duty cycle for “positive” (a) and “negative” (b) 2D DSE structures. The duty cycle is defined as the ratio of the width of the individual feature (W in Figure 9a) forming the 2D DSE to the grating period d:

DC=Wd.

The following shifts parameters were used in the simulation: ΔX = ΔY = 1.737 µm for “positive” structure and ΔX = ΔY = 1.729 µm for “negative” structure. The grating period d did not change and was equal to 5 µm. In the investigated range DC = 0.14 µm−0.26 µm (W = 0.7 µm–1.3 µm), the simulation results for a “positive” 2D DSE written on a chromium film 50 nm thick show that the DE of the 0th diffraction order decreases from ~89.7% to ~75.3%. For “negative” 2D DSE, a similar increase in the structure size leads to an increase in the diffraction efficiency of the 0th diffraction order from ~2.8% to ~6.0%. In this case, the efficiency of the 0th DO is practically independent on the centers shift for the gratings that form the 2D DSE structure relative to each other and is less than 0.2% in the entire simulated range of shifts ΔX and ΔY. Therefore, the 0th order is not informative for determining the shifts when writing CGH, but its measurement can be used to control the duty cycle of the 2D DSE structures. In addition, Figure 19 shows that a change in the duty cycle of 2D DSE structures leads to a shift in the equilibrium point of the diffraction efficiency. Due to this, there is a redistribution of energy between 1st and 2nd diffraction orders and, as a consequence, a change in the ∆*DE*_12_ parameter. Thus, a change in the duty cycle from the designed value leads to an error in the proposed method determining the shifts during CGH writing. It is important to notice than DC is function of the feature size and also the period which can slightly change due to discretization effects and local errors of writing beam positioning. To control the duty cycle of the formed 2D DSE structures and determine the initial displacement of the point of the equilibrium diffraction efficiency (caused by a possible change in the feature size), it is possible to form one more reference 2D DSE structure with precisely specified displacement values ΔX and ΔY before the start of CGH writing. The measured values *DE*_0_ and ∆*DE*_12_ for this 2D DSE structure can be used as a reference for all other 2D DSE structures which are intended for determining the shifts when writing the CGH.

Implementation of the proposed CGH inspection method based on built-in diffractive sensor elements requires automating the search for DSEs in the CGH pattern and measuring the diffraction efficiency for them in the 1st and 2nd diffraction orders. This task can be performed on the basis of, for example, an optical diffractometer described in [39]. The search for DSE marks can be organized by defining the coordinates of the registration marks on the CGH in the coordinate system of a two-axis motorized stage of the diffractometer.

In addition to automating the process of measuring the diffraction efficiency, the development of the proposed method is supposed to be carried out in the direction of both studying its resistance to deviations in the geometric dimensions of the structure, and increasing the spatial resolution by reducing the writing spot diameter and, as a consequence, the feature size W. It is also important to study the features of applying the method to binary-phase CGHs.
